# Structural insights into the career path between pre- and postgraduate physician-scientist training programs

**DOI:** 10.7554/eLife.87148

**Published:** 2023-10-02

**Authors:** Christopher S Williams, Emily J Gallagher, Don C Rockey, Olujimi A Ajijola, Patrick J Hu, Barbara I Kazmierczak, Christopher D Kontos, Jatin M Vyas, Mone Zaidi, Kyu Y Rhee

**Affiliations:** 1 Department of Medicine, Vanderbilt University Medical Center Nashville United States; 2 Department of Medicine, Icahn School of Medicine at Mount Sinai New York United States; 3 Department of Medicine, Medical University of South Carolina Charleston United States; 4 Department of Medicine, University of California, Los Angeles, Geffen School of Medicine Los Angeles United States; 5 Department of Medicine, Yale University School of Medicine New Haven United States; 6 Department of Medicine, Duke University Medical Center Durham United States; 7 Department of Medicine, Massachusetts General Hospital Boston United States; 8 Department of Medicine, Weill Cornell Medicine New York United States; Augusta University United States; Augusta University United States

**Keywords:** physician scientist, PSTP, training, career

## Abstract

The growing complexities of clinical medicine and biomedical research have clouded the career path for physician-scientists. In this perspective piece, we address one of the most opaque career stage transitions along the physician-scientist career path, the transition from medical school to research-focused internal medicine residency programs, or physician-scientist training programs (PSTPs). We present the perspectives of medical scientist training program (MSTP) and PSTP directors on critical features of PSTPs that can help trainees proactively align their clinical and scientific training for successful career development. We aim to provide both trainees and MSTP directors with a conceptual framework to better understand and navigate PSTPs. We also offer interview-specific questions to help trainees gather data and make informed decisions in choosing a residency program that best supports their career.

Physician-scientists play an irreplaceable role in the biomedical workforce (National Institutes of Health, 2014), yet they frequently navigate a career path that is conspicuously deficient in vocational structure. The unpredictable pace and direction of scientific progress often serve as an excuse for the absence of structured career development and training pathways for nascent physician-scientists. However, the emergence of physician-scientist communities across disciplines and career stages, such as the American Physician Scientist Association (APSA), American Society for Clinical Investigation, Association of American Physicians, Physician Scientist Support Foundation, and others, has recently begun to challenge this view (Daye et al., 2015; Hurley et al., 2020; Utz et al., 2022; Williams et al., 2022; Jain et al., 2019).

Career stage transitions can be stressful and confusing points along a physician-scientist’s career path, both for trainees and their mentors. Yet, focused discussions with established physician-scientists have consistently revealed striking similarities in their career paths across a range of clinical and scientific disciplines. Such conversations have identified gaps in training and mentorship, as well as opportunities to define a more structured and streamlined career path (National Institutes of Health, 2014; Williams et al., 2022; McElvaine et al., 2021; Milewicz et al., 2015). Here, we focus on the first of these transitions, from medical school to research-focused internal medicine residency programs, commonly referred to as physician-scientist training programs (PSTPs). In this perspective, we seek to address common and important misperceptions associated with this transition.

We offer the independent but shared perspectives of medical scientist training program (MSTP) directors and PSTP directors on critical features of PSTPs owing to the current absence of systematically collated data or information about this topic. While we hope that this piece will inspire broader, more formal surveys and studies on this topic, we hope that these perspectives will immediately empower trainees to be more purposeful and proactive in aligning scientific training, clinical training, and career development during residency and fellowship. Rhetorical questions, followed by explanations of their significance and responses, are intended to provide trainees with a conceptual framework for understanding and navigating PSTPs (Figure 1). We then reformulate these questions in a manner more directly useful to trainees during the interview process.

**Figure 1.**
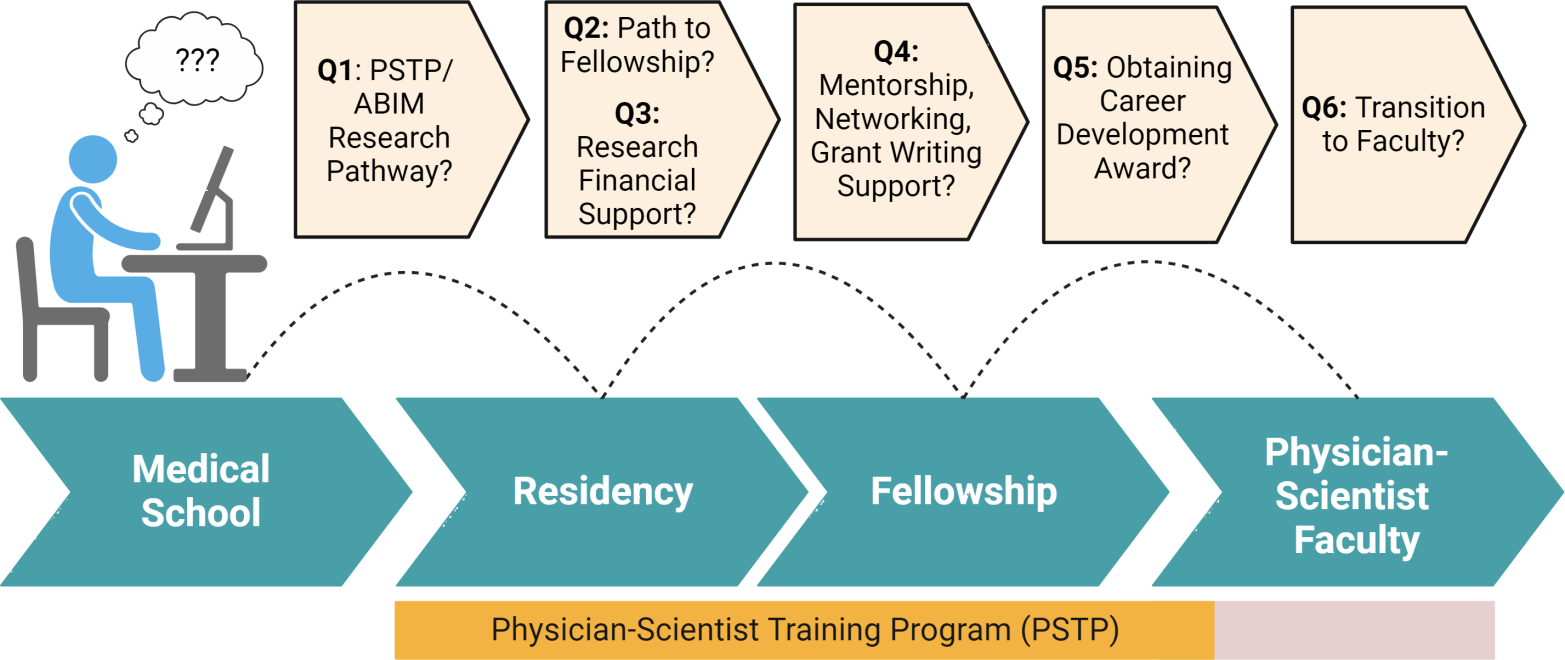
Key questions about support during critical career development stages. A number of important considerations, which are discussed in detail in the text, that are important in physician scientist career planning. Created with BioRender.

## Question #1

- Isn't "PSTP" just a name used to describe a residency program that follows the American Board of Internal Medicine (ABIM) Research Pathway?
  - The ABIM Research Pathway is a clinical credentialing mechanism that enables trainees to consolidate clinical training in internal medicine and (optional) subspecialty fellowship with 36 mo of dedicated research training time (80% protected research effort). This mechanism allows trainees to achieve board eligibility for certification in internal medicine and (optionally) a clinical subspecialty while expanding protected research time. Details of this pathway and specific guidance about clinical requirements for even advanced subspecialty training (e.g. interventional cardiology, electrophysiology, advanced GI endoscopy, etc.) are available online at the ABIM website. Moreover, these requirements provide a regulatory framework around which clinical training programs and trainees can develop cohesive research and career development plans. The ABIM Research Pathway thus facilitates physician-scientist career development but is often insufficient as a stand-alone mechanism for career development. PSTPs are often linked to the ABIM Research Pathway, and many PSTPs offer the ABIM track within their program, but this is not always the case.
  - In contrast, PSTPs are postgraduate physician-scientist career development programs that provide structured training and community. Such training includes coaching in career-specific research skills, individualized mentorship, financial support, and, most importantly, a community of physician-scientist peers and mentors to enhance career development. PSTPs can promote diversity, equity, and inclusion in physician-scientist careers, particularly when the community of PSTP trainees and faculty as well as institutional leadership are aligned with this goal. This includes being aware of your peers and the programs available for career development as they can vary among institutions and have an impact on the research faculty available. A list of PSTPs with website links was collated by the APSA and is currently being developed for redeployment as a web resource.
  - It is important to appreciate the distinctions between PSTPs and programs that offer only the ABIM Research Pathway. First, it may help trainees assess how much formal experience programs have with physician-scientist training. Questions about the specific administrative support mechanisms that are (or are not) in place for ensuring regulatory compliance with the ABIM are useful here. Second, and more importantly, it may help distinguish programs best able to develop trainees into independent investigators from those that simply ensure board eligibility. Finally, it may help trainees identify the programs best equipped to meet their specific scientific goals within the framework of a clinical training environment.
  - From an administrative perspective, while the ABIM Research Pathway does not exclude foreign medical graduates, not all PSTPs, or residencies, for that matter, are able to accept foreign medical graduates due to institution-specific visa sponsorship policies. Such policies are of particular relevance to PSTP programs owing to the mixed and extended clinical and research training requirements of the ABIM Research Pathway.
  - Importantly, the ABIM Research Pathway offers a regulatory framework that can be referenced when defining a trainee’s clinical and research activities with clinical training program directors and administrators.

## Question #2

- Do all PSTPs provide a direct path to clinical fellowship and protected research time?
  - Despite the specific requirements of the ABIM Research Pathway for board eligibility, PSTPs exhibit considerable variation in administrative organization, flexibility, and support. Some PSTPs span departments and disciplines and function on an institutional level, while others are localized within departments or disciplines. Key areas of variation include the following (each point is followed by key questions to consider):
    - Mechanisms and level of fellowship commitments (both within and outside of the same institution). While the ABIM Research Pathway does not require that residency and fellowship training occur at the same institution, many programs offer some form of assurance of a fellowship position in the PGY3 year of their PSTP.
      - How and when do these assurances occur and with what level or form of assurance?
      - Do they extend to advanced subspecialty fellowships such as in cardiology and gastroenterology?
      - What types of administrative and programmatic support are available to facilitate this process?
      - What flexibility is there in choosing or changing subspecialty fellowship preference, both in relation to time and choice of subspecialty and location?
    - Structure and flexibility of clinical and research activities during fellowship
      - Is there a guarantee of salary support at or above the postgraduate year (PGY) pay scale during the research-intensive phase of fellowship? (See Question #3 for related questions.)
      - What are the clinical responsibilities/expectations during the research phase of fellowship training?
      - Some programs may be structured in a way that research time has interspersed blocks of clinical training. What is the longest continuous block of research time protected from clinical responsibilities (not including the ABIM-mandated half day/week continuity clinic) during fellowship? This is a particularly important consideration for those pursuing wet bench work where extended periods of uninterrupted time are often essential.
      - Is there flexibility to frontload or customize the clinical training component?
      - What sort of flexibility is provided for trainees who have family responsibilities arise during this training period? What are the policies on parental/caregiver leave should the need arise?
    - Integration with PSTP during fellowship
      - Who bears primary responsibility for career development during the fellowship phase of training?
      - Is there continuous oversight from a centralized PSTP, or is there handoff of this responsibility to the fellowship program?
      - Are there regular and structured programming activities and/or mentorship committees explicitly focused on career development?
    - Range of accessible mentors and labs
      - What scientific and career mentors are available?
      - Is there a system in place to create structured mentorship dyads and/or mentorship teams, coaches, and sponsors?
      - What resources are available for identifying mentors and determining the postdoctoral training record and funding history of potential mentors? Does the institution provide an avenue for identification of a mentor during the interview process, even before matching?
      - What limitations or restrictions are there on the choice of lab? Are trainees limited to working in the labs of department/division faculty, or are they free to choose from labs across the institution, or even at other institutions?
      - What policies are in place regarding mentors if the relationship doesn’t work?
  - PSTPs also vary in the roster of faculty with track records of training physician-scientists who have successfully transitioned to independent scientific careers. Trainees interested in clinical subspecialty training should become familiar with the track record of the relevant fellowship training program vis a vis physician-scientist trainees, as well as that of the subspecialty Division Chief and/or Fellowship Program Director who are often involved in reviewing applications and/or the interview process.
  - Perhaps the most important source of variation among PSTP-associated fellowship programs is the cohort of available scientific mentors. Trainees are advised not to select an institution based on a single faculty member whose research aligns with their interests as interests may evolve and faculty may move. However, it is critical to be certain that the institution has and will have appropriate mentorship available during training. Candidates are further advised to learn whether their own research questions and approaches of interest are valued by PSTP-affiliated faculty. Useful resources for current research interests and funding include departmental and laboratory websites, the NIH RePORTER database (https://reporter.nih.gov), and the Blue Ridge Institute for Medical Research annual report, which depicts institutional and departmental NIH funding levels (https://brimr.org) OR (https://brimr.org/brimr-rankings-of-nih-funding-in-2022/).
  - For trainees in an ABIM Research Pathway who pursue fellowship training at an institution different from that where they complete their residency, there may be variability in the familiarity of the relevant fellowship program director and institution with the requirements of the ABIM Research Pathway as well as their ability to support external trainees.
  - Whether staying within an institution for residency and fellowship or moving elsewhere, open discussion between the residency and fellowship program directors is essential to ensure optimal integration of clinical and research training. This is best achieved through coordinated communication of Accreditation Council for Graduate Medical Education, National Institutes of Health (ACGME) milestones among the residency and fellowship program directors and the trainee to ensure adequate preparation for certification in Internal Medicine after the first clinical year of subspecialty fellowship training as well as certification in the subspecialty upon completion of ABIM Research Pathway requirements.
  - A critical element of a PSTP is its ability to guarantee continuous protected research time during the fellowship phase of training. This perspective is often lost in relation to clinical responsibilities. The practical importance of this protected time cannot be overstated. First, and most importantly, this period is the major, if not sole, window of opportunity for trainees to lay the experimental foundation for their future independent scientific careers. Prior research training, whether as an MD-PhD student or research fellow during medical school, is critical for the development of scientific thought processes. Nonetheless, trainees usually pursue research in a different area during the postgraduate phase, from which they transition to their long-term careers. Second, protected research time often comes to a hard stop that usually coincides with completion of the ACGME-accredited duration of clinical fellowship training. This time constraint is rarely a factor for PhD investigators, whose postdoctoral training period is more flexible and often substantially longer than the time afforded by the ABIM Research Pathway. Third, this period of training must usually result in objective demonstrations of productivity (i.e. publications and grant applications/awards) that often require substantial lead time for preparation and/or revision. As a result, the actual time available to achieve these metrics is functionally shorter than the time allotted during training, leading to late-stage attrition of physician-scientists who run out of time to meet these milestones. However, it is relatively common for trainees to negotiate a transition to a non-tenure track faculty position including continued protected research time (see Question #6).

## Question #3

- Do all PSTPs guarantee salary support for the 3 y of research training required by the ABIM Research Pathway?
  - Despite the administrative requirements of the ABIM, PSTPs vary in their sources and mechanisms of support for research training, which can result in different levels and durations of guaranteed salary support. Below, we provide some questions that will help clarify the specific assurances and expectations at different programs.
  - The first, and perhaps most straightforward question should be, ‘Is my salary guaranteed at the institutional PGY pay scale during the research phase of training?’ This will allow applicants to distinguish programs that answer ‘yes’ from those that provide any other response. More detailed follow-on questions include:
    - What, if any, dedicated financial resources are available to ensure salary support during the research-intensive phase of training, or is salary evaluated on a case-by-case basis?
    - Which fellowships have a T32 and/or other funding sources to support fellows in their research training, and are other resources available to ensure equity on a PGY pay scale (i.e. supplementing T32 funding above the NIH scale)?
    - What, if any, support is there for trainees interested in pursuing an NIH F32 or equivalent award as part of their training in preparation for a K-level, or equivalent, career development award?
    - What limitations or restrictions are associated with any such funding support, including, but not limited to, choice of labs, duration of support, or other administrative or scientific obligations?
  - Equally important questions include the following:
    - What support/resources do programs offer for transitioning to faculty or other positions *after* finishing the ABIM Research Pathway portion of training?
      - For those requiring additional research training time:
        - What types of non-tenure track positions are available, and what responsibilities are associated with these positions, whether clinical or otherwise?
        - What internal and external funding mechanisms are available, if any, to provide support after completion of training? These might include internal career development awards (CDAs) that provide funding while the junior faculty member is applying for external career development awards such as the NIH K-mechanism CDAs, Veteran’s Administration CDA, or Foundation/Society CDAs.
    - What types of resources/support are available to facilitate transition to bona fide tenure-track faculty positions?
  - Institutional support for trainees in the research years of fellowship and beyond to the point of securing a career development award, such as an NIH K08, K23, or K99 grant, or foundation-based equivalent, is essential. Trainees should thus understand the available mechanisms of support, conditions and/or limitations associated with that support (e.g. choice of lab or mentor), duration of support, and other administrative, clinical, or scientific obligations.
  - Trainees should be aware of the opportunity to apply for loan repayment programs designed to pay down debt for trainees pursuing research-based careers. Consistent with the goal of supporting research-based careers, paylines on these mechanisms tend to be high. These mechanisms can further offset financial pressures that might derail one’s career. Examples include the NIH Loan Repayment Program (https://www.lrp.nih.gov).
  - For non-US graduates, who have historically comprised some 16% of ABIM research pathway graduates (Lipner et al., 2012), knowledge of available visa sponsorship mechanisms is of equal practical importance. This is not only for legal reasons but also for its impact on the range of potential support mechanisms (federal vs. foundation) that trainees might be eligible for, both at the fellowship and career development stages. Because the research phase of some PSTPs is funded through NIH T32s, some PSTPs are not able to support non-US graduates.

## Question #4

- Do PSTPs offer more than access to clinical fellowships, scientific mentorship, and financial support?
  - A strength of well-established PSTPs is that they typically incorporate specific career development activities, individualized individual development plans (IDPs), and career mentorship into their curricula. Some questions to ask in this regard include the following:
    - What resources and/or activities are in place to help identify an appropriate mentor(s) and research project?
    - Are there formal career mentorship committees?
    - What types of resources are available to facilitate work–life balance?
      - Are there resources/mechanisms to assist with spousal employment, child care, or elder care?
  - Having a mentorship committee and/or advisors is important for the career development of physician-scientists, and such committees are required as part of a future career development award. Assembling such a committee early in one’s research training facilitates guidance during the research phase and is a critically important contribution of the PSTP. Mentorship committees need not be static but may change over time. Early in the clinical training, the residency program director and PSTP director may play central roles in this committee, while over time the primary mentors will transition to the fellowship program director and research mentor along with the PSTP director. An external committee member may also help ensure the trainee is publishing and developing research independence in order to advance their career as well as provide critical scientific advice. In addition to mentors, trainees also often benefit from including sponsors and coaches who can help address more specific scientific and career development issues.
  - Well-established programs have close and active relationships with institutional individuals and teams who offer resources, networks/communities, and programs, including those that provide skills in career development (e.g. grant writing, *see below*), leadership, responsible conduct of research, and diversity, equity, and inclusion.
  - Well-established programs often have a repository of previously successful grant applications, will conduct workshops to help develop projects and grant applications, and will identify senior faculty or near-peer faculty who can also provide advice on a proposal, particularly in its early stages of development. Other career development workshops may include topics such as managing teams, budgets, conflicts of interest and commitment, and promotions.

## Question #5

- What are the success rates and timelines for obtaining a career development award (CDA, NIH K award, or equivalent) across programs?
  - Are there programs that provide guidance and assistance specifically for assembling CDAs?
    - Does the program have grant banks, grant writing workshops or classes, and/or mock study sections?
  - It is axiomatic to say that grants are the currency of academic career development. Objective measures of grant funding are thus an essential, though admittedly imperfect, metric of PSTP productivity. As some PSTPs are relatively young, metrics of grant productivity may not yet represent the training and potential of recent alumni. However, in the context of the physician-scientist career path, the time-to-first-CDA is an emerging metric of program success. This is an increasingly important issue as trainees often face new and complex work–life balance issues during this period of their lives.

## Question #6

- What happens after completion of the ABIM Research Pathway component of a PSTP?
  - PSTPs are often viewed by institutions as an internal pipeline for future faculty recruits. However, opportunities available for trainees to transition to faculty positions differ among institutions and may vary depending on whether or not a trainee has secured a CDA or are close to doing so.
    - Trainees who have already secured a CDA at or near the time of formally completing a PSTP are highly competitive (and often actively recruited) for a faculty position on a national level and are poised to begin independent tenure track careers at the Assistant Professor level.
    - Trainees who have not yet secured a CDA or published their primary work typically require additional mentored support and often transition to non-tenure track faculty positions, such as Instructor or equivalent. Because trainees on this pathway have not yet attained the credentials typically indicative of scientific independence, these faculty appointments are often fraught with a diverse range of conditions and expectations that require careful negotiation.
      - Particularly important considerations in this case are the amount of funded, protected research time; expected clinical and administrative responsibilities; and future opportunities for funding and lab space following acquisition of a CDA or equivalent. A particularly important consideration with respect to funded protected research time is the availability of formal post-PSTP ‘bridge’ mechanisms/programs which even when present often vary in both programmatic content and financial support.
      - A closely related issue is the need to avoid inadvertently starting the tenure clock with an Assistant Professor appointment before funding is obtained.

## Question #7

- What does ‘success’ look like for a graduate from your program?
  - A graduate of a PSTP:
    - Has a deep understanding of the scientific method and is able to apply it in research.
    - Possesses strong analytical skills and can effectively identify the root causes of problems.
    - Has a track record of successful research projects and publications in top-tier academic journals.
    - Is able to collaborate effectively with interdisciplinary teams, including scientists, physicians, and other healthcare professionals.
    - Has excellent communication skills and can effectively communicate complex scientific concepts to both scientific and lay audiences.
    - Is able to secure research funding from a variety of sources, including government agencies and private foundations.
    - Is committed to advancing scientific knowledge and improving patient care through research and innovation, and finally.
    - Is well prepared for a successful career in research and may be employed in academia, industry, or the federal and state governments and a substantial amount of time is devoted to discovery-based activities.

We aim to provide prospective applicants with a clearer understanding of the complex environment surrounding PSTPs. For this purpose, we have compiled a concise list of essential questions summarizing the concepts discussed above. These questions will prove valuable for trainees during their residency interviews in a PSTP.

### Specific questions applicants might consider asking during a PSTP interview

- Does your program offer a letter-of-intent for specific clinical fellowships and/or an accompanying 3-year assurance of protected research time?
- What mentors are available, and what has their track record in career development been?
- Can I work in any lab I choose within the institution?
- Does your program provide guaranteed salary support on the institutional PGY pay scale throughout the period of protected research time?
- How is the required 20% clinical time during the research phase of training structured? Do I take call? Could I be conscripted for ‘backup’ clinical coverage?
- What other resources/support does your program provide for career development?
- Beyond salary/stipend support, does your program provide research funding during the research training phase?
- How is your program addressing the challenges and opportunities associated with diversity, equity, and inclusion?
- What sort of programming/resources are provided at the end of PSTP training for transitioning to faculty (for those staying at your institution)?
- Tell me about recent outcomes and graduates from your program.
